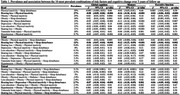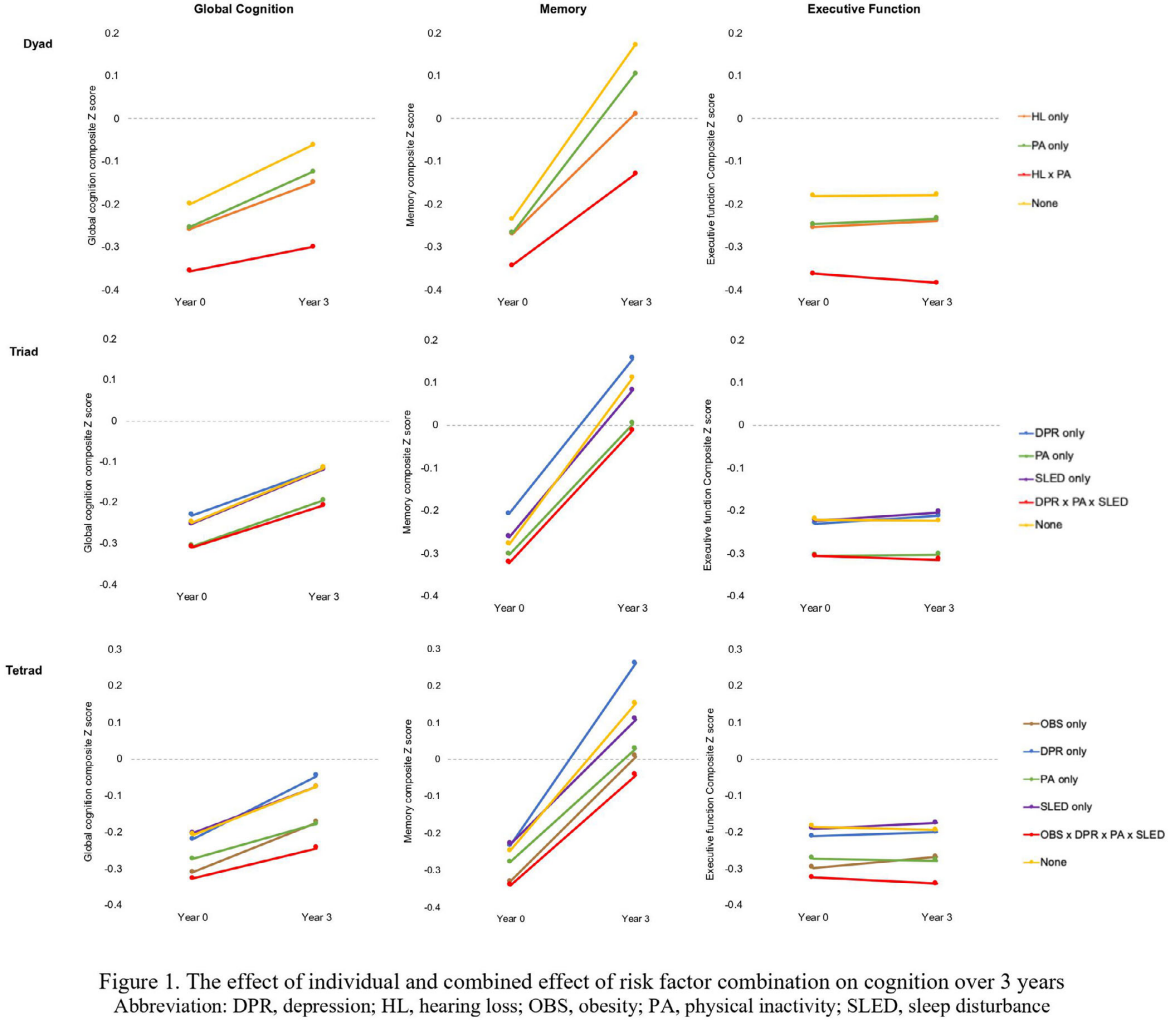# Identifying the optimal combinations of dementia risk factors to be targeted in multidomain lifestyle intervention in Canada

**DOI:** 10.1002/alz.092069

**Published:** 2025-01-09

**Authors:** Surim Son, Mark R. Speechley, Guangyong Zou, Manuel Montero‐Odasso

**Affiliations:** ^1^ Schulich School of Medicine & Dentistry, Western University, London, ON Canada; ^2^ Schulich School of Medicine & Dentistry, Division of Geriatric Medicine, Western University, London, ON Canada

## Abstract

**Background:**

The optimal combinations of modifiable risk factors to be targeted in preventive dementia trials may vary across countries and settings. We aimed to identify the combinations of modifiable risk factors associated with cognitive change in Canadian adults.

**Method:**

Population Attributable Fraction analyses on 30,097 participants from the Canadian Longitudinal Study on Aging and prevalence of 2, 3 and 4 risk combinations of the 12 modifiable risk factors identified in the 2020 Dementia Lancet report were estimated to note the ten most prevalent combinations. Association between the identified combinations and 3‐year cognitive changes was examined with linear mixed models.

**Result:**

Risk factor combinations were associated with greater change in memory than executive function (Table 1). Among the ten most prevalent dyad combinations, hearing loss and physical inactivity showed the largest adjusted effect on global cognition (β = ‐0.08, 95% CI ‐0.09 to ‐0.06), memory (β = ‐0.1, ‐0.18 to ‐0.14), and executive function (β = ‐0.03, ‐0.04 to ‐0.02). The triad of hearing loss, physical inactivity, and sleep disturbance was associated with the largest adjusted effect across all domains (global: β = ‐0.06, ‐0.07 to ‐0.04; memory: β = ‐0.12, ‐0.15 to ‐0.09; Executive Function: β = ‐0.03, ‐0.04 to ‐0.01). The tetrad of hearing loss, depression, physical inactivity, and sleep disturbance was associated with the largest adjusted effect on memory (β = ‐0.11; ‐0.18 to ‐0.05). The dyad that was associated with greater memory change than their individual effect was hearing loss and physical inactivity, while it was depression, physical inactivity, and sleep disturbance for the triad combination (Figure 1). The tetrad combination included obesity, depression, physical inactivity, and sleep disturbance.

**Conclusion:**

Identifying risk factor clusters with the highest prevalence and potential effect size can optimize efficiency of trial design. Intervention programs should consider including hearing loss and physical inactivity or obesity, depression, physical inactivity, and sleep disturbance. These findings may help strategically tailor multidomain dementia intervention programs to have the greatest impact in the Canadian population.